# Dual targeting of toll-like receptor 4 and angiotensin-converting enzyme 2: a proposed approach to SARS-CoV-2 treatment

**DOI:** 10.2217/fmb-2021-0018

**Published:** 2021-02-11

**Authors:** Laura Kate Gadanec, Tawar Qaradakhi, Kristen Renee McSweeney, Benazir Ashiana Ali, Anthony Zulli, Vasso Apostolopoulos

**Affiliations:** ^1^Institute for Health & Sport, Victoria University, Melbourne, Australia

**Keywords:** ACE2, angiotensin-converting enzyme 2, coronavirus, coronavirus disease 2019, COVID-19, SARS-CoV-2, TLR4, toll-like receptor

The occurrence of the SARS-CoV-2, the virus responsible for COVID-19, has rapidly become a pandemic, resulting in devastating effects to the global economy and public health [1], with over 70.4 million cumulative cases and 1.6 million deaths [2]. While the majority of infected patients remain asymptomatic or experience mild symptoms, the pathophysiological hallmarks of the severe form of COVID-19 is characterized not only by viral infection, but accompaniment of an exacerbated and dysfunctional inflammatory response, which has been correlated to systemic immunopathological consequences (i.e., cytokine storm), pulmonary and respiratory complications, multiorgan failures and mortality [1,3,4]. Despite current knowledge pertaining to the pivotal role that angiotensin-converting enzyme 2 (ACE2) has during SARS-CoV-2 infection, burgeoning evidence is emerging, reporting viral susceptibility in intra- and extrapulmonary immune and non-immune cells lacking or expressing low concentrations of ACE2 [5–7]. Furthermore, loss of ACE2 activity during SARS-CoV-2 infection may result in augmented pulmonary injury and worsened outcomes in patients with pre-existing cardiovascular comorbidities [7,8]. Additionally, recent publications have revealed toll-like receptors (TLR), specifically TLR4, as major contributors to SARS-CoV-2 infectivity and pathogenesis [1,9]. The SARS-CoV-2 S protein has been shown to favor and directly bind to the extracellular domain of TLR4 [1], which may result in the elicitation of the aggressive inflammatory response observed in patients with severe COVID-19. As the developments of vaccines and pharmaceutical treatments to target SARS-CoV-2 are underway, researchers and clinicians have been challenged to urgently create an acceptable therapy to control infection and improve patient outcomes [1,10]. We propose that a multipronged pharmaceutical treatment targeting restoration of ACE2 activity and TLR4 inhibition may represent an appealing approach to combat SARS-CoV-2 virus.

## Angiotensin-converting enzyme 2

ACE2, a component of the renin-angiotensin system, has gained notoriety by being identified as a key receptor involved in SARS-CoV-2 S protein entry into host cells [11–13]. However, burgeoning evidence is emerging, reporting viral susceptibility in intra- and extrapulmonary immune and non-immune cells lacking or expressing low levels of ACE2 [5–7]. Additionally, the theory associating enhanced infection susceptibility, severe pathological outcomes and increased mortality rate in patients with cardiovascular disease comorbidities prescribed pharmaceuticals that indirectly increase ACE2 expression [14–16] was unfounded during a recent clinical trial [16]. The randomised 30-day clinical trial was conducted to determine if continuing ACE inhibitors and angiotensin II receptor blockers (n = 325) versus suspending treatment (n = 334) placed COVID-19 patients previously diagnosed with pre-existing cardiovascular diseases at a greater risk to SARS-CoV-2 viral susceptibility and worsened patient prognosis [16]. Results from the trial determined that these therapies failed to enhance intracellular viral load and had no effect on the occurrence of severe COVID-19 outcomes or mortality rate [16]. Yet COVID-19 patients with pre-existing cardiovascular disease have a five times higher mortality rate than those without [8].

A previous letter, authored by our team, outlined the potential use of diminazene aceturate (DIZE) (an ACE2 activator [17]) as a treatment to restore ACE2 activity in COVID-19 patients [18]. Patients with severe COVID-19 experience organ dysfunction, which manifests as acute respiratory syndrome and acute cardiac, hepatic and renal injury [19]. Damage to multiple organ systems may be caused by loss of ACE2 function subsequent to SARS-CoV-2 binding [7,8]. ACE2 downregulation has previously been associated with myocardial [20] and pulmonary disease [21] in humans. In murine models of influenza A, proper homeostatic functioning of circulating ACE2 has been shown to be protective against influenza A-induced acute lung injury [22]. Conversely, ACE2 deletion in mice has been associated with increased aggravation and severity during influenza A infection [22]. Therefore, we postulate that ACE2 may have an undetermined protective role during SARS-CoV-2, which is silenced/lost once SARS-CoV-2 enters and hijacks host cells. Thus, restoring ACE2 functions through administration of DIZE may promote cardiovascular and pulmonary protection against SARS-CoV-2 and may improve COVID-19 patients (with and without pre-existing comorbidities) outcomes.

## Toll-like receptor 4

Our hypothesis of alternative receptors involved in SARS-CoV-2 infection is supported by current literature demonstrating interactions between TLRs and the S protein of SARS-CoV-2 [1]. TLRs are integral to innate immunity, as they are sentinel pattern recognition receptors responsible for host surveillance by identifying foreign- and self-molecular signatures [23,24]. Ten functional TLRs have been reported in humans, which are abundantly expressed in immune and non-immune tissues, including cardiac, pulmonary, renal, hepatic and nervous systems [25]. TLRs display specialty and specify by identifying unique pathogen-associated molecular patterns (highly conserved motifs displayed by pathogens) [23] and danger-associated molecular patterns (endogenous alarm signals released by stressed, damaged or dying host cells), independent of infection [24]. However, the end product of robust sterile inflammation, produced through the MyD88-dependent pathway (TLR1, 2, 4–10) [26] or the TRIF-dependent pathway (TLR3 and 4) [27], is ubiquitous among TLRs independent of the origin of the activating ligand. Recent literature has identified TLR4 as a key mediator during SARS-CoV-2 infection, involved in both infectivity [1,28] and induction of the vicious inflammatory response [29] reported in patients with severe symptoms. While TLR3 and 7 agonists have been proposed as potential therapeutic targets for prophylactic agents to prime antiviral innate immune system responses [30–32], their ability to engage with SARS-CoV-2 S protein and to be used for pharmaceutical targets after infection has been established remains elusive. Therefore, due to the involvement of TLR4 during infectivity and establishment of infection, we postulate that its inhibition may provide a promising treatment to combat COVID-19.

TLR4 is predominantly responsible for providing immunity against Gram-negative bacterial, through identification of lipopolysaccharides [33,34]. However, association and subsequent activation of TLR4 by viral fusion proteins and glycoproteins, including viruses that target the pulmonary system [35,36], have been reported. An *in silico* study, investigating the TLR-binding efficacy to SARS-CoV-2 S protein, demonstrated direct engagement between TLR1, 4 and 6 and subunit 1 of SARS-CoV-2 S protein [1]. Of which, TLR4 was favored, displaying the highest binding efficacy value of -120.2 [1]. The results from this study have been supported by a preprint indicating direct binding between the trimeric S protein of SARS-CoV-2 and TLR4 in human monocytes (THP-1 cell line) [28]. Therefore, TLR4 may represent an alternative pathway used by SARS-CoV-2 to gain entry into cells, accounting for viral vulnerability reported in intra- and extrapulmonary immune and non-immune cells lacking or expressing low concentrations of ACE2 [5–7].

Uncontrolled TLR4-mediated inflammation has been suggested to contribute to immunopathological consequences in COVID-19 patients. Peripheral blood mononuclear cells harvested from COVID-19 patients demonstrated increased expression of TLR4 and its corresponding downstream signaling molecules (CD14, MyD88, TRAF6, IRAK1, TIRAP, TICAM-1 and NF-kB) [29]. Additionally, patients infected with SARS-CoV-2 have increased levels of activating danger-associated molecular patterns of TLR4, including β-defensin-3 [37], fibrinogen [38,39], HSP70 [40], HMGB1 [41], syndecan [42], S100A8/9 [29,41,43], and surfactant A and D [44]), which may be responsible for inducing persistent and aggressive inflammation, resulting in cytokine storm and severe pulmonary dysfunction. This is supported by reports of patients with COVID-19 displaying increased levels of cytokines and chemokines releases after TLR4 activation (IL-1β, -2, -6, -8, -9, TNFα, G-CSF, GM-CSF, MIP-1α and MIP-1β [45,46]).

Taken together, we postulate that inhibiting TLR4 using the novel antagonist resatorvid (TAK242) may improve patient outcomes by preventing systemic infection and dampening the inflammatory response. TAK242 is a potent and selective TLR4 antagonist, which displays anti-oxidant and anti-inflammatory abilities [47]. It is believed that TAK242 is able to exert its potent anti-inflammatory abilities due to its low molecular weight, allowing for rapid distribution to sites of inflammation [48]. Our hypothesis is supported by results demonstrating the ability for TAK242 to potently inhibit release of IL-1β from human THP-1 cells after exposure to SARS-CoV-2.

## Conclusion

As there has yet to be significant advances in vaccine development against the SARS-CoV-2 vaccine, there is great urgency to develop an effective pharmaceutical treatment to be administered to patients to improve outcomes. We propose that a dual-acting pharmaceutical treatment, which combines TLR4 inhibition and ACE2 activation, may represent a strategic therapeutic approach to dampen severe inflammation, restore ACE2 functionality, protect cardiac and pulmonary tissues and inhibit SARS-CoV-2 infection. While the development of a universal vaccine remains underway, a combination therapy of DIZE (ACE2 activator) and TAK242 (TLR4 inhibitor) may be a promising therapy to be administered to patients, allowing for effective treatment of COVID-19 patients.
